# Cognitive analytic therapy for self-harm with young people: protocol for the RELATE-YP feasibility randomized controlled trial

**DOI:** 10.1186/s40814-026-01880-0

**Published:** 2026-07-14

**Authors:** Peter Taylor, Isabel Adeyemi, Martin Eden, Samantha Hartley, Mark Hann, Cameron Latham, Sarah Parry, Catherine Robinson, Stephen Kellett

**Affiliations:** 1 Division of Psychology & Mental Health, School of Health Sciences, University of Manchester, Manchester Academic Health Sciences Centre, Manchester, UK; 2 Pennine Care NHS Foundation Trust, Manchester, UK; 3 Manchester Centre for Health Economics, The University of Manchester, Manchester, UK; 4 Rotherham, Doncaster, and South Humber NHS Foundation Trust, Doncaster, UK

**Keywords:** Self-harm, Young people, Clinical trial, Cognitive analytic therapy, Feasibility

## Abstract

**Background:**

Self-harm is a major health concern amongst young people that is increasing in prevalence. Cognitive analytic therapy (CAT) is a relational, integrative psychotherapy that is theoretically consistent with our growing understanding of self-harm. The aim of this study was to ascertain the feasibility and acceptability of evaluating brief CAT in young people aged 13–17 who self-harm using a randomised controlled trial (RCT) design.

**Method:**

Sixty young people with experiences of self-harm will be recruited from child and adolescent mental health services. Participants will be randomly allocated (1:1 ratio) to brief CAT plus treatment as usual (TAU) or TAU alone. Assessors will be blind to treatment allocation. Assessments will occur at baseline, 8, 16, and 20 weeks after randomisation. A qualitative process evaluation will investigate experiences of the therapy and trial procedures. The primary outcome for this study is feasibility, judged against a priori progression criteria relating to recruitment, retention, adherence to therapy, and missing data.

**Discussion:**

Brief CAT may help young people struggling with self-harm, but research is needed. In order to undertake a definitive RCT, it is first necessary to resolve a number of feasibility uncertainties. The planned feasibility trial will help inform a larger definitive trial focused on the efficacy of brief CAT for young people who self-harm.

**Trial registration:**

The trial was pre-registered (01/08/25) on ISR CTN (ISRCTN code: ISRCTN11505839).

**Supplementary Information:**

The online version contains supplementary material available at https://doi.org/10.1186/s40814-026-01880-0.

## Introduction

Self-harm, encompassing acts of self-injury and overdose irrespective of suicidal intent [1], often starts during adolescence with self-harm amongst young people increasing in recent years [2–6]. A meta-analysis of global self-harm rates in children and adolescents suggests a lifetime prevalence of 14% [7]. The occurrence of self-harm behaviour is associated with an increased risk of future self-harm and suicide, as well as physical and mental health difficulties [8–11]. Whilst there is evidence that psychological approaches can help young people who self-harm, this evidence remains scarce [12, 13]. Effective intervention with young people who self-harm may prevent further occurrence and escalation of these difficulties. Given the growing pressures facing child and adolescent mental health services (CAMHS; [14]), brief intervention holds the promise of filling gaps in service provision and enabling more rapid access to therapy.

One potential brief therapy that could be helpful for young people who self-harm is brief cognitive analytic therapy (CAT). Cognitive analytic therapy (CAT; [15]) is a time-limited, relational therapy, which has already been investigated as an approach for young people with complex difficulties [16–18]. brief CAT has been developed as an 8-session approach and already investigated in adults who self-harm [19]. However, there are currently no trials of brief CAT for young people who self-harm. CAT is usually 16 or 24 sessions long. The 8-session format of brief CAT was guided by the recognised value of shorter interventions in reducing waiting times and the additional benefit to young people in fitting around education and other commitments.

CAT focuses on identifying unhelpful relational patterns that underlie presenting difficulties. The therapist then helps the client to better recognise these patterns and find alternative, more adaptive ways of being and responding [15]. Interpersonal processes and context play an important role in self-harm [20]. Qualitative research also suggests that self-acceptance and forging more positive relationships with others aid recovery from self-harm [21, 22]. CAT is able to work with these relational processes and so may be well suited to helping people who self-harm. A recent meta-analysis of 25 studies provides support for CAT but studies to date predominantly focussed on adults [23]. Case series of brief CAT-informed approaches in young people (13–17 years) or are in eating disorder services (15–24 years) support the acceptability of this approach [16, 18], with positive feedback and good attendance rates. A randomised controlled trial (RCT) in young people [15–18] meeting criteria for sub-clinical or full borderline personality disorder comparing CAT to good standard care reported a faster reduction in internalising and externalising symptoms in the CAT arm [17].

National guidelines currently recommend cognitive behavioural therapy (CBT) and, where emotion-regulation difficulties are present, dialectical behaviour therapy (DBT) as treatments [1]. Whilst there is support for these approaches, it remains limited [12, 13]. For example, a meta-analysis reported small treatment effects and noted effects across therapies were smaller in young people [13]. DBT is an intensive approach involving multiple components including groups, parental involvement, one-to-one therapy, and telephone coaching. Such intensive interventions may not always be suitable or necessary for all young people and so alternatives are needed [24]. Both CBT and DBT have a largely behavioural focus. CAT, as a relational therapy, if found to be effective, may provide a valuable alternative that enhances patient choice. Consequently, brief CAT for self-harm warrants further investigation, and if found to be effective would help improve the range of treatment options in services.

Prior to evaluating the efficacy of brief CAT for young people who self-harm, it is important to determine the feasibility of investigating this approach within an RCT design. A feasibility trial can help answer important feasibility uncertainties and identify barriers and challenges that can help inform the design of a larger efficacy trial [25]. A recent feasibility trial has supported the feasibility and acceptability of brief CAT for adults who self-harm [19]. However, CAMHS is a very different context to adult services, and young people require different adaptations and considerations as part of psychological therapy.

RELATE-YP is a feasibility RCT that will evaluate the feasibility and acceptability of brief, 8-session CAT for young people (13–17 years) who self-harm. This age range has been chosen to coincide with when self-harm often first occurs [3]. brief CAT will be compared against treatment as usual (TAU). TAU has been selected as a comparator because no single approach is currently consistently implemented for young people who self-harm within UK services. Feasibility will be judged based on pre-specified progression criteria regarding recruitment rates, retention at follow-up, and missing data. Given finite healthcare resources, decision-makers require evidence demonstrating the cost-effectiveness of healthcare interventions. We will therefore also assess the feasibility of collecting the necessary data for a full economic evaluation of the intervention within a future trial. Acceptability will be investigated via a qualitative process analysis and therapy adherence rates. Adverse events (AEs) will be monitored as part of the study. Treatment effects on clinical outcome measures (e.g. self-harm behaviour and urges) will be estimated as this will help guide the design of a future definitive trial by informing key parameters (e.g. variance in outcome measures). This protocol has been drafted based on version II of the protocol (19/8/2025), in accordance with the updated Standard Protocol Items: Recommendations for Interventional Trials (SPIRIT) statement (see Supplement I; [26, 27]). The results of this feasibility trial will directly inform future clinical research into treatments for self-harm, determining if a definitive trial of brief CAT for self-harm is feasible, and if so, guiding the design and development of this deinfitive trial. A definitive trial would in turn contribute to clinical guidelines regarding effective treatments for self-harm in young people.

## Method

### Design, randomisation, and blinding

**Fig. 1:**
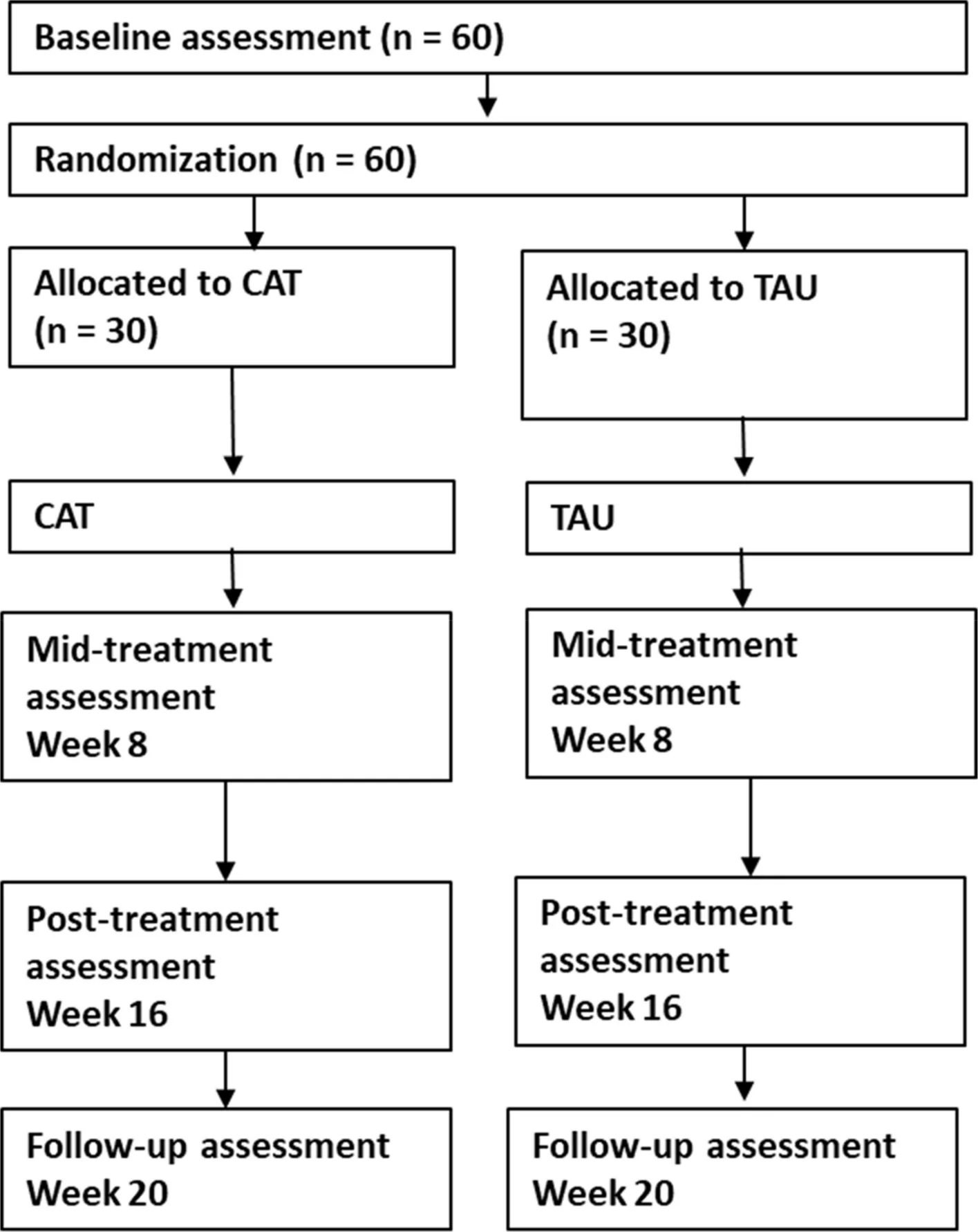
Flow diagram of study procedures

A single-blind feasibility RCT design will be undertaken whereby participants are randomly allocated on a 1:1 basis to either brief CAT plus TAU or TAU alone. Assessments will take place at baseline, 8-, 16-, and 20 weeks post-randomisation. Randomisation will take place after the baseline assessment and be conducted independently using SealedEnvelope.com, using a randomised block design (block sizes of 4 and 6 generated at random) that is stratified by site (NHS trust). Allocation concealment will be ensured by independent randomisation, with researchers involved in enrolling participants and assessment not having access to randomisation sequence. Members of the research team not blind to allocation will inform participants of their allocation. A flow chart of trial procedures is available in Fig. 1.

Researchers completing assessments will be blind to treatment allocation. To help adherence to the study protocol, participants, families, and clinical teams will be briefed on the importance of blinding. Blinded researchers and trial therapists will not share offices. Blinding will be monitored throughout and blind breaks recorded. In the event of a break in blinding, an alternative researcher who is still blinded will undertake future assessments for that participant, where possible. The statistician will be blind to participant allocation status up until the creation of the statistical analysis plan (SAP) and its approval by the trial steering committee (TSC). Blinding may be broken where required to manage risk. Blinding of participants is not possible, as is typical in psychotherapy trials, because participants will know if they are receiving brief CAT. Whilst this increases the potential for performance bias, we will attempt to minimise bias overall by following best practice for trials of psychological therapies, such as using valid assessments, taking steps to reduce attrition, and carefully monitoring what other interventions participants access.

### Trial setting and participants

The trial will take place across three large and demographically diverse NHS Trusts in the North of England. Participants will be young people with experiences of self-harm who will be recruited through CAMHS and associated services (e.g. mental health in schools’ teams, other outpatient psychology services for young people).

Participants will be required to meet the following inclusion criteria:

- Aged 13–17 years.
- Engaged in self-harm, following the NICE definition [1], two or more times in their lifetime, with at least one episode in the past year. This will be confirmed via the Self-Injurious Thoughts and Behaviours Interview Short Form (SITBI; [28]).
- Can be safely seen in an outpatient clinical context in which treatment is being provided as judged by their clinical team or referrer.

Exclusion criteria will be as follows:

- Moderate-to-severe intellectual disability (IQ: < 70) as judged by a young person’s clinical team.
- Organic cerebral disease/injury affecting receptive and expressive language comprehension as judged by a young person’s clinical team.
- Currently an inpatient.
- Currently experiencing an active episode of mania or psychosis, assessed with the Mini International Neuropsychiatric Interview for Children and Adolescents (MINI-KID; [29]).
- Judged at imminent risk of harm to themselves, operationalised as presence of intent or planning to end their lives within the near future (e.g. next 2 weeks). In such cases, individuals could still participate once the level of risk has declined.
- Currently receiving another active one-to-one psychological therapy. This would include structured psychological therapies such as CBT or DBT. Other forms of support, like a support group or skills group, seeing a clinician for informal support or medication advice, or engaging with self-help materials, will not exclude people.

### Recruitment and consent

Services will be asked to identify potentially eligible young people, share information about the trial, and determine whether they are happy to be contacted by the research team. For young people aged under 16 years, a parent or guardian will also need to agree to them being contacted by the research team. The service can then refer the young person to the study team. Clinical services (e.g. CAMHS) may identify potentially eligible young people at various points including through review of waiting lists and service records. Referred young people will be screened by a researcher for eligibility and, if eligible and interested in participating, invited to take part in a baseline assessment. Baseline assessment meetings will either be in person or online via video call, depending on practicability and preference. Informed consent would be taken at the start of the baseline assessment meeting by a researcher (who will receive training in consent taking). For those aged under 16 years, consent from a parent or guardian along with assent from the young person would be sought, whilst only consent from the young person will be required for those aged 16 or 17 years. If the meeting is in person, then a physical consent form will be used. If the meeting is occurring online, then an audio-recorded verbal consent process will be used whereby a researcher takes the participant (and their parent or guardian if required) through a consent script and records their responses on an encrypted device. The study team will contact and share information about the study with local schools, colleges, and CAMHS within the recruitment area, raising awareness of the study. We will focus on contacting services and institutions in socioeconomically deprived areas and demographically diverse areas in particular, to try and encourage participation from these locations, given that health inequalities can affect participation in research trials [30].

### Baseline characteristics

During the baseline assessment participant demographic information (age, gender, ethnicity) and clinical history (past and current physical and mental health diagnoses, receipt of psychological therapy, and medication use) will be recorded in order to characterise the sample. Sexual orientation will also be recorded if known, given that self-harm rates are elevated amongst sexual minority groups [31, 32]. We will also record socioeconomic deprivation based on current postcode using the Indices of Multiple Deprivation [33], as this is another important risk factor for self-harm. The MINI-KID [29] will be used to determine mental health presentation. This is a structured interview that assesses past and current mental health difficulties. Only the modules relating to depression, common anxiety disorders (panic disorder, social anxiety, generalised anxiety disorder), bipolar disorder, and psychosis will be used to minimise burden.

### Primary outcomes

The primary trial outcomes relate to feasibility and acceptability. Data on recruitment rates, participant retention at follow-up assessments, missing data, and adherence (i.e. attendance at therapy sessions) will be recorded and evaluated against a set of pre-specified progression criteria (see Table 1). These criteria will indicate whether progression to a definitive trial is warranted, using a traffic light system (green = progress to larger trial; amber = modification needed; red = do not progress). Acceptability will also be evaluated via the qualitative process analysis. Levels of missing data and qualitative data will guide decisions about the most appropriate primary outcome for an efficacy trial. It is anticipated that self-harm urges, assessed via the Alexian Brothers Urges to Self-Injure scale (ABUSI; [34]), or behaviour, assessed via the Self-Injurious Thoughts and Behaviours Interview Short Form (SITBI; [28]), would be the primary outcome in a subsequent trial. However, the suitability of these outcomes would be evaluated based on statistical considerations and participant feedback plus guidance from the Patient and Public Involvement (PPI) advisory group.

**Table 1:** Progression criteria

Outcome	Criterion	Green	Amber	Red
Recruitment	Ability to randomise 60 participants in a 12-month recruitment window	≥ 80%	60%—79%	< 60%
Retention	Percentage of participants attending the 16-week assessment as the potential primary outcome timepoint	≥ 75%	60%—74%	< 60%
Outcome Suitability	Missing data on candidate primary outcomes (SITBI, ABUSI) at 16-week assessment	≤ 15%	16%—25%	> 25%
Adherence	Percentage of participants receiving the minimum dose of therapy (≥ 4 sessions) within 16-week treatment window	≥ 75%	60%—74%	< 60%

### Clinical outcomes

A range of assessments of clinical outcomes will be taken, covering self-harm behaviour and urges, depressive symptoms, perceived recovery, internalising and externalising problems, and perceived dependence on self-harm. Table 2 presents a summary of the timing of study assessments. Data will be checked for missing data, and floor and ceiling effects for these measures which may affect their inclusion in a larger trial. Participants’ experience of completing measures, ascertained from the qualitative process evaluation, will also inform decisions.

**Table 2:** Overview of assessment schedule

Measure	Baseline	8 weeks	16-weeks	20-weeks
ABUSI	x	x	x	x
AEP				x
Bespoke service use measure	x			x
CHU9D	x			x
ESIQ	x	x	x	x
MINI-KID	x			
PHQ-A	x	x	x	x
ReQuestYP	x	x	x	x
SDQ	x	x	x	x
SITBI	x	x	x	x

*ABUSI* Alexian Brothers Urges to Self-Injure scale, *AEP* Adverse Experiences in Psychotherapy questionnaire, *CHU9D* The Child Health Utility Instrument, *ESIQ* The Experiences of Self-Injury Questionnaire—Positive Beliefs subscale, *MINI-KID* Mini International Neuropsychiatric Interview for Children and Adolescents, *PHQ-A* The Patient Health Questionnaire—9 for adolescents, *ReQuestYP* The Recovery Questionnaire, *SDQ* Strength and Difficulties Questionnaire, *SITBI* Self-Injurious Thoughts and Behaviours Interview

#### Self-harm behaviour and urges

The SITBI short form [28] is a structured interview measure that will be used to assess suicide attempts and non-suicidal self-injury (NSSI). Sections of the measure will be adapted in line with the revised version of the SITBI [35] to improve the clarity of questions. Specifically, the opening question about NSSI will use the wording from the revised SITBI that provides additional detail on what might be considered as NSSI and some examples of behaviours that would not count as NSSI including indirect forms of harm (e.g. starving oneself). The ABUSI [34], a 5-item questionnaire, will be used to assess urges to self-harm over the past week. Scores range from 0 to 30 with higher scores indicating more severe urges. The validity and reliability of both measures have been demonstrated with young people [28, 34].

#### Dependence on self-harm

The Experiences of Self-Injury Questionnaire—Positive Beliefs subscale (ESIQ; [36]) is an 8-item questionnaire that measures positive beliefs and perceived dependency on self-harm. Scores range from 0 to 32 with higher scores indicating greater dependency. Research has supported the factor structure, validity, and reliability of this measure [36].

#### Depressive symptoms

The Patient Health Questionnaire—9 for adolescents (PHQ-A; [37, 38]) is nine-item self-report questionnaire that assesses depressive symptoms in young people over the past 2 weeks. Scores range from 0 to 27 with higher scores indicating worse symptoms. The PHQ-9 has been recommended as a screening measure for depression in young people with acceptable sensitivity and specificity [39]. The PHQ-A includes some adjustments to wording to make the measure more appropriate to young people [37, 38].

#### Internalising and externalising problems

The Strength and Difficulties Questionnaire self-report version (SDQ; [40, 41]) is a 25-item self-report questionnaire that assesses a range of difficulties in young people including internalising, externalising, and peer problems. A total difficulties score can be generated which ranges from 0 to 40, with higher scores indicating greater difficulties. The factor structure and validity of the SDQ have been supported and total score has good internal consistency [41].

#### Perceived recovery

The Recovery Questionnaire (ReQuestYP; [42]) is a questionnaire that assesses perceived recovery from mental health difficulties over the past week. Scores range from 0 to 90, with higher scores indicating greater recovery. The reliability and validity of the measure have been supported [42, 43].

### Health economics

The feasibility of collecting data necessary for a full economic evaluation will be assessed through the collection of service-use data and health-related quality of life (HRQL). The cost of providing the intervention and TAU will be estimated based on therapist-recorded service-use during the trial. A bespoke resource use questionnaire will be developed based on the adolescent Client Service Receipt Inventory (CSRI; [44, 45]) to capture details of other health and social care service utilisation. The Child Health Utility Instrument (CHU9D; [46]) is a nine-item measure of HRQL for 7- to 17-year-olds. Utility weights can be assigned to CHU9D descriptive health states and can then be used to generate quality-adjusted life years for use in economic evaluation. These measures will be completed at baseline and 20 weeks.

### Brief cognitive analytic therapy

Brief CAT will be delivered across eight 50–60 min sessions, typically on a weekly basis, following an established 8-session protocol [15, 47] that has been written to reflect the standard tri-phasic reformulation, recognition, and revision CAT approach. The therapy will focus on self-harm but will also seek to identify and work with the underlying processes that contribute to self-harm, including relational roles and patterns. Sessions 1–2 focus on *reformulation*, where a shared understanding of a young person’s difficulties is developed. Session 3 is where the narrative reformulation is delivered (i.e. start of the recognition phase). Sessions 4–5 further focus on *recognition*, where a young person is supported to better notice unhelpful patterns and roles in their day-to-day life. Sessions 6–8 then focus on revision, where ‘exits’ from unhelpful patterns and roles are identified and practised. Throughout CAT, there is a focus on the therapeutic relationship, including the potential enactment of patterns here that could occur elsewhere in a young persons’ life. A follow-up session is arranged approximately 4 weeks following the final session to help consolidate therapy gains and problem solve remaining issues.

Sessions will take place either in person or online via video call. Therapy will be delivered by a qualified psychological therapist (e.g. clinical psychologist, counselling psychologist) at band 7 or 8 who has completed CAT practitioner training. Supervision will be provided at least fortnightly by a CAT accredited supervisor and CAT psychotherapist. Fidelity will be assessed by rating a random 10% subset of sessions using the Competency of Cognitive Analytic Therapy measure (CCAT; [48]) by an independent expert. The CCAT is widely used for determining the competency of CAT. An adherence checklist will also be kept by therapists and reviewed in supervision. Supervision will also involve listening to recordings of sessions to ensure adherence and competency.

Participants will be asked to complete sessional tracking forms at each session that focus on the recognition and revision of idiographic target problems (TPs) and target problem procedures (TPPs). These tracking forms are used as part of the therapy to guide and focus the work taking place. These forms will be collated and analysed to investigate idiographic change amongst participants receiving brief CAT. Participants in the brief CAT arm will be asked to complete the session rating scale [49], a measure of perceived working alliance, at the end of therapy.

### Treatment as usual

TAU will follow standard care provided by CAMHS. There is often no standard pathway for young people who self-harm. Instead, the level and nature of input varies depending on factors such as risk and perceived need. TAU might commonly include:

- Assessment and formulation with a clinician
- Risk assessment and management with a case manager or partnership worker
- One-to-one work on emotion regulation (this may be using a non-standardised model)
- Group emotion regulation skills sessions
- Structured psychological therapies (e.g. CBT, DBT) as recommended by NICE
- Family therapy/systemic family practice

A patient would rarely get offered all of these, and higher-intensity interventions such as CBT, DBT, or family therapy are less likely to be offered. Within the trial, participants in both groups will be able to continue to access medication, support groups (that do not adopt a structured psychological model), and safety planning (including meetings with a case manager or partnership worker), and psychiatric support as routinely provided by services. For participants allocated to the brief CAT arm of the trial, it would be clinically contraindicated to offer another high-intensity, 1-to-1, talking therapy that occurs at the same time as the brief CAT sessions, however. Access to health and social care services and interventions will be carefully monitored as part of the trial. We will record what TAU typically involves, using the bespoke service use measure described above, which will inform the development of a health economic workstream for the definite trial. We will not be offering any additional care following each participant’s involvement in the trial.

### Data collection

Assessment sessions will take place with a researcher either in person or online via video call or telephone. In person sessions might take place in a participant’s home, school, CAMHS offices, university, or other NHS building. Allowing flexibility in the modality of assessments may aid engagement and retention. Researchers will administer questionnaires and interviews and support the young person to answer questions as needed. Data will be collected via a case report form (CRF). A copy of the CRF, excluding copyrighted material, is available on request. Researchers will receive training in delivering assessments and regular, at least fortnightly, supervision.

Participants and services will also receive a newsletter that will update them on news about the trial and thank them for their involvement. This may aid engagement and retention, especially amongst those in the TAU arm, but helping them to feel a part of the trial between contacts. Participants will be free to withdraw from the trial at any time. If they are in the brief CAT arm, they may choose to end therapy early but continue with the research components of the trial. Where participants choose to leave therapy or the trial early, they will be asked for their reasons for leaving, to aid us in identifying possible barriers to engagement.

### Qualitative process evaluation

The qualitative process evaluation will enable an in-depth investigation of trial acceptability and the identification of potential therapy mechanisms of action. Qualitative interviews with stakeholders including clinicians and parents are also planned as part of a linked, parallel study. Participants from both arms of the trial will be invited to take part in qualitative interviews (*n* = 15 from brief CAT arm; *n* = 5 from TAU arm). A larger subsample from the brief CAT arm will be sought to allow more exploration of perceived therapy impact and mechanisms or action. Sample size targets have been guided by the principles of information power [50]. Given the relatively narrow focus of the study, the specific population of interest, and the expectation of in-depth data based on similar studies [51, 52], a sample of 20, which is common for process evaluations of this nature, has been chosen [19, 53].

Participants will be sampled from the main trial with their consent. Maximum variation sampling will be used to create a diverse sample with regard to trial retention (i.e. whether people withdraw early or not), gender, age, neurodiversity, and ethnicity. This will be achieved by inviting participants sequentially to be interviewed and monitoring the characteristics of this subsample as it develops, so that the choice of the next person to be invited can be informed by these characteristics. A semi-structured schedule will guide interviews. The interview schedule will be developed with people with lived experience involved and guided by the theoretical framework for acceptability of healthcare interventions [54]. The interview schedule will be piloted. Interviews will be audio recorded on an encrypted device to aid transcription. Interviews are expected to last up to 60 min and could take place in person or remotely via video call.

The researcher undertaking qualitative interviews will have experience of qualitative research and will have received training in qualitative methods. They will not have any past relationship with participants. The lead qualitative researcher will have a PhD in psychology or a related discipline. The Consolidated criteria for reporting qualitative research (COREQ; [55]) will be used when reporting qualitative results.

### Trial management

PT and SK are co-chief investigators and will hold overall responsibility for trial management. A monthly trial management meeting will include all co-investigators and will be the key forum for decision-making. A weekly operational management meeting will support day-to-day running. A trial steering committee (TSC) consisting of clinicians, researchers, experts-by-experience, and a statistician will meet at least bi-annually. This will provide independent oversight on the trial. TSC members will be independent of the trial sponsor or funder. Given the small scale of this trial, no clinical trials unit will be involved, and no separate data monitoring and ethics committee (DMEC) will be set up. Instead, a subgroup of the TSC will take on the role of the DMEC, including monitoring adverse events (AEs) within the trial. The research team will provide the TSC with regular (at least quarterly) updates on trial progression, AEs, and challenges, to facilitate ongoing monitoring.

### Patient and public involvement

The trial has been designed with the involvement of young people with lived experience of self-harm, and those with lived experience will continue to be involved in the running of the trial. The trial team includes a co-investigator with lived experience of self-harm and a research assistant with lived experience. These individuals will contribute to management meetings and ensure the perspective of those directly affected by self-harm is taken into account. A PPI advisory group consisting of young people with lived experience of self-harm will also meet four times a year, providing guidance and informing decision-making during the trial.

### Safety monitoring and reporting

AEs and serious adverse events (SAE) will be monitored and recorded throughout the trial. SAEs will include events that result in death, are life-threatening, or result in hospitalisation. This will therefore include some but not all instances of self-harm. AEs related to self-harm behaviour and ideation, interpersonal conflict, and deterioration in mental health are expected. The research team will review all SAEs to determine the probability that the event is causally linked to study procedures or interventions based on available data (e.g. timing of event and participant report on antecedents). The TSC will have access to SAE reports and will provide independent monitoring of SAEs throughout the trial. The TSC will also monitor the overall number of AEs and SAEs occurring during the course of the trial. An imbalance in AEs and SAEs between trial arms is expected due to factors such as random variation and the increased participant contact for those in the brief CAT arm (i.e. AEs may be more readily identified due to the regular appointments). Any substantial imbalance will require a review.

Therapy may be discontinued in cases where this is required to maintain the safety of participants or others. Where SAEs are attributed to the trial procedures, this may be grounds to discontinue therapy or withdraw a participant from the trial altogether. A participant may be withdrawn if the research team are notified of a significant potential threat to the safety of a member of the research team or if a participant displays aggressive or abusive behaviour towards a member of the team. These decisions would be made in consultation with the participant’s clinical team, and the TSC, and would occur on a case-by-case basis. Where SAEs are attributed to the trial procedures, it may also be necessary to pause or stop the trial altogether, and such decisions will be made with the TSCs and the funder’s guidance.

At the 20-week assessment, participants will be asked to complete the Adverse Experiences in Psychotherapy questionnaire (Hutton P, Byrne R, Morrison A: Adverse Effects in Psychotherapy (AEP) measure, unpublished). This is a self-report questionnaire designed for trials of psychological interventions. Participants are asked about a range of adverse experiences that may arise from taking part in a trial of a psychological intervention. Total scores are not generated for this measure but instead items scored as greater than ‘a little’ (i.e. scores ≥ 3) are treated as substantive.

### Sample size

We will aim to recruit *N* = 60 participants (30 per trial arm). This number will enable key outcomes of interest, linked to our progression criteria, such as retention rates, and key parameters to inform a power calculation for a larger trial, such as the standard deviation of clinical outcome measures, to be estimated with adequate precision and minimal bias [56, 57]. For example, Teare and colleagues [56] recommend a sample of 60 or more based on simulation results for estimating event rates. Note that we are interested in event rates across both arms for our progression criteria (i.e. we are not evaluating progression criteria separately for each arm).

### Data management

Participant data will be stored confidentially and anonymised upon completion of the trial. Participant data will be stored securely either in locked cabinets at NHS sites or on secure, password-protected NHS computer drives. Identifiable information will be stored separately to other study data, using a linking code to connect the two types of data. Datasets will be designed to minimise data entry errors by having pre-specified admissible values. A 10% subset of data entry will be done independently, in parallel, to assess quality of data entry. Discrepancies will be reviewed and further parallel data entry undertaken where data entry problems are noted. A detailed study data management plan is available from the authors on request. Anonymised trial data will be made available to other researchers following publication of the main trial paper, via a suitable online repository.

### Statistical analysis

As this is a feasibility study, we will report descriptive data on study ‘processes’ and ‘outcomes’. An intention-to-treat (ITT) protocol will be followed. Descriptive data concerning recruitment, retention, and attrition will be presented in a CONSORT flow chart [58]. Treatment engagement and attrition rates will be calculated with associated confidence intervals and reasons for leaving the trial or therapy early will be reported where available. Rates of missing data will be presented for outcome measures. Descriptive statistics will be presented for the overall sample and by group for all potential outcome measures. RELATE-YP is a feasibility study and inferential testing of treatment efficacy is therefore not the goal of the study. Nonetheless, we will estimate treatment effects and associated confidence intervals to inform planning for a future trial. These analyses will use an appropriate regression model, depending on the distribution of the outcome variable (e.g. linear regression for continuous outcomes, negative binomial regression for count outcomes). A detailed SAP will be drafted by the trial statistician and approved by the TSC prior to any analyses taking place. This will be available from the research team on request. No interim analyses are planned. Imputation may be used to generate total scores on scales where < 20% of items are missing but will not otherwise be used.

For the feasibility economic analysis, the extent of missing cost and outcomes data will be presented using descriptive statistics. The CHU9D will be used to derive quality-adjusted life years (QALYs), in line with National Institute for Health and Care Excellence (NICE) reference case [59]. The cost of delivering the intervention will be estimated based on the resources used as recorded by trial therapists. Published NHS and social care unit costs will be attached to these estimates of resource use. The identity and quantity of other health and social care resources used by participants will be captured and summarised. This information is required for a full economic evaluation and will be used to understand what services participants access as TAU to help define exemplar comparators for a future cost-effectiveness analysis. Data on what participants received as TAU will be presented in our final reporting and reviewed holistically to help inform the planning of a future definitive trial.

Each participant in the brief CAT arm will rate a range (1-3) of TPs and associated TPPs at each session, including the follow-up. Participants therefore will generate a time-series of sessional TP/TPP recognition and revision scores. To analyse changes in recognition and revision ratings, linear mixed-effects models will be used. Models will then be extended to include random slopes if this improves model fit. Follow-up session TP/TPP ratings will not be included in the mixed-effects model, because of the time interval between the final CAT session and the follow-up (i.e. 4 weeks). Trajectories of predicted revision and recognition TP/TPP scores will also be plotted. A more detailed analysis plan with be pre-registered prior to undertaking these analyses.

### Qualitative process analysis

Qualitative data concerning acceptability of the intervention will be transcribed verbatim and analysed via framework analysis [60], which is useful for research with multi-disciplinary teams, clinicians, patients, and the public to evaluate trial delivery, implementation, and participant experience. We will follow the qualitative framework analysis approach [60, 61]. The steps of the analysis will comprise: (1) data familiarisation, (2) identifying a thematic framework by developing a coding scheme, (3) indexing all study data against the framework, (4) charting to summarise the indexed data, and (5) mapping and interpretation of patterns found within the charts. Members of the advisory group will be invited to contribute to the analysis and provided with training to enable this. Qualitative data regarding participant’s view on the acceptability of trial procedures more generally will be analysed using content analysis, generating themes based on shared ideas and perspectives. NVIVO software may be used to assist analysis [62]. Anonymised participant quotes will be presented as part of the results.

### Ethics

This trial is currently under review from an NHS research ethics committee (Greater Manchester Central; REC ID: 344,320). Any proposed modifications to the trial that require ethical amendments will be reviewed by the TSC and sponsor.

### Dissemination

Trial results will be disseminated through peer-reviewed journal articles and via presentations at national and international conferences. Press releases may be issued to coincide with the publication of results and to share the results of the trial amongst the general public. A variety of stakeholders including clinicians, young people, trial participants, parents and guardians, and researchers will be invited to a dissemination event once the trial is completed, where the results will be presented and discussed.

## Discussion

Self-harm can have a major impact upon a young person’s life, often being a source of substantial distress and a robust risk factor for later difficulties including further acts of self-harm. Given the increasing rates of self-harm amongst young people, it is clear that evidence-based psychological therapies are needed. Brief CAT has potential as a brief and structured relational therapy that may complement and add to existing treatment options for young people that self-harm. As a relational therapy, CAT differs to existing behavioural approaches like DBT and CBT, placing greater emphasis on interpersonal processes including the therapy relationship, and seeking to understand self-harm in terms of underlying relational patterns. Brief CAT may provide a helpful alternative, expanding service provision and enhancing patient choice. CAT has a developing evidence base in young people but clinical trials are needed to determine its efficacy as a treatment for difficulties like self-harm in young people [63, 64]. The RELATE-YP trial is therefore an important first step in evaluating brief CAT for young people who self-harm, and will answer important feasibility uncertainties, that in turn will inform the development of a larger, definitive trial. For example, data regarding recruitment rates and attrition will inform the required length and sample size of a future study, whilst qualitative data may lead to refinements to the therapy or trial procedures. The proposed feasibility trial, given its sample size, is not suitable for testing efficacy, but by paving the way for a larger definitive trial will help to inform clinical practice and guidance regarding treatments for self-harm in young people.

## Supplementary Information

Supplementary Material 1.

## Data Availability

An anonymised version of the final trial dataset will be made available to other researchers following publication of the study.
